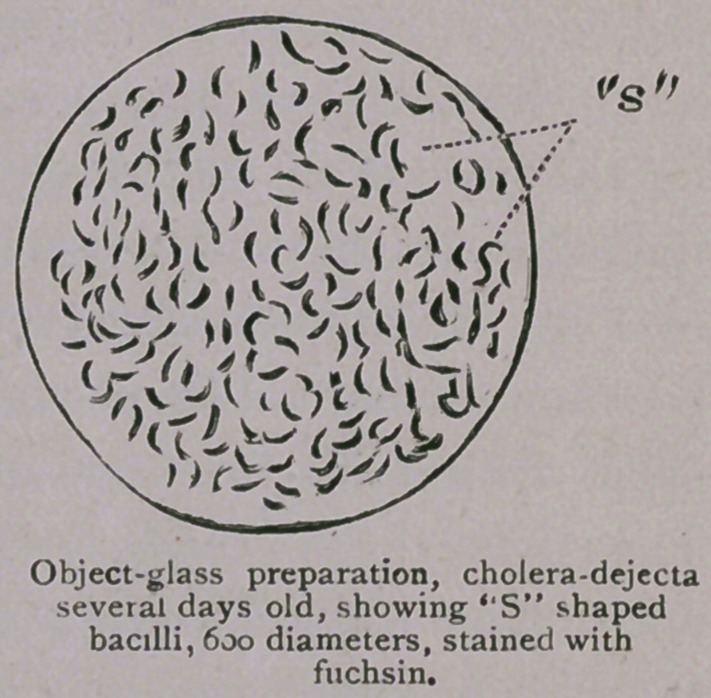# Dr. Koch’s Cultivation Experiments in Cholera Bacillus

**Published:** 1885-03

**Authors:** George W. Lewis

**Affiliations:** Berlin


### Dr. Koch's Cultivation Experiments in Cholera Bacillus.

By George W. Lewis, Jr., A. B.

On October 1, 1884, the German government completed arrangements with Dr. Robert Koch by which he was to establish, in the city of Berlin, a laboratory well equipped with apparatus and assistants for the purpose of acquainting the German physicians with his theory of the cause of cholera, and the mode of cultivation of the so-called "comma-bacillus." The time specified for the duration of this course was to extend from October 1, 1884, to the end of January, 1885, and the four months were to be divided into periods of ten days each, that time being sufficient for a thorough understanding, not merely of the theory, but also of the practical work necessary in the cultivation and detection of the cholera bacillus. Delegations from the principal cities and towns were to be received in groups of from four to six at a time, and all their work was to be under the direct supervision of Dr. Koch, aided by a competent corps of assistants, most of whom have accompanied him during his investigations in Egypt, Italy and France. At the expiration of the ten days allotted to a single group, another was to take its place and receive a similar course.

Some two weeks ago I had the good fortune to receive one of the two appointments granted to Americans, and should like to give the reader some idea of the thoroughness and practicability of such instruction. Before doing this, however, a few words with regard to the present understanding of the disease may not be entirely out of place. Perhaps no visitation of the epidemic has afforded a better opportunity for studying its characteristics and tendencies than the recent outbreak in Italy and France, and for this reason most of our knowledge comes from these sources. The idea that cholera is of spontaneous origin is now no longer entertained by those who have given particular attention to the subject. Dr. Koch, who is undoubtedly the oldest investigator in this direction, is of opinion that its home is in the Delta of the Ganges, and his reasons for thus closely confining its limits seem at least plausible. The conditions that favor the spread of cholera in India are of a very peculiar nature. It was formerly maintained that the disease was indigenous in Ceylon, Madras and Bombay, but later research indicates that the almost constant existence of the infection in these places in due to the active traffic between them and certain parts of the Delta. The only region, however, in India where the cholera prevails continuously and without apparently any fluctuation, is the Delta of the Ganges. This entire tract is the unceasing home of the epidemic. It even extends up the banks of the Ganges as far as Benares. The upper part of the Delta is densely inhabited, while the lower part or base of the triangle is unapproachable to man on account of the the inundations and pernicious fevers which invariably attack any one who passes its borders. In this uninhabited district may be found a luxuriant vegetation and an abundant variety of animal life, and one can easily imagine what quantities of animal and vegetable matter are here exposed to putrefaction. As Dr. Koch maintains, there is perhaps no better place in the world for the development of micro-organisms, and especially micro-organisms of an infectious character. In this respect the boundary between the inhabited and uninhabited parts of the Delta would seem to be exceptionally favorable, where the refuse from an extremely thickly populated country is floated down by the small streams, and mixes with the brackish water below, which flows backwards and forwards and is already saturated with putrefied matter.

The theory that the comma-bacillus belongs to a special fauna and flora of micro-organisms whose growth and development are adapted to these surroundings, is very probable, for everything points to the fact that cholera derives its origin from this frontier territory. This statement may appear more valuable when we consider that all the greater epidemics have been accompanied by a corresponding increase of the disease in the south of Bengal. We now know that the comma-bacillus finds, in the districts adjoining the supposed habitat, the most favorable conditions for obtaining a footing and transferring itself from man to man. The entire stretch of country known as Lower Bengal is only slightly raised above the sea-level, and during the rainy season almost the whole extent is submerged. For this reason the inhabitants are compelled to build their huts upon raised ground. This is effected by taking the earth near where the hut is built in order to raise the ground on which the house stands. The result of thus displacing the earth is to leave a large tank adjoining each hut in which soiled water and putrefied matter from the household rapidly collect. Strange as it may seem this very water is used for drinking and other household purposes, and in turn receives much of the refuse matter which is necessarily thrown out. Under these circumstances can it be wondered at that the deadly cholera germ should take its origin and be transferred from one to another until it reaches all Europe and America?

In the first place, the differential diagnosis between cholera Asiatica and cholera nostras is by no means apparent from the clinical presentation of the disease, nor can they be distinguished with any degree of certainty from cases of acute arsenic poisoning. For these and other less obvious reasons, it is extremely difficult to tell, from a single case, whether it is really cholera or the result of poisoning; and when a new part has become infected, the physicians have found themselves in hot dispute as to whether, after the first suspicious case, the strictest sanitary measures should be enforced. In this way the most precious time is consumed, and the cholera germ, if it proves to be such, has gained a wide-spread circulation.

Through the discovery of the cholera bacillus, which has received the very characteristic name of "comma" bacillus, a speedy diagnosis is rendered possible. In spite of all opposing assertions, this characteristic biological and microscopical bacillus is found in no other infection save cholera, and by means of

Dr. Koch's simple yet comprehensive method of "pure culture," every physician would be able to detect the existence of the organism with perfect certainty. The possibility of thus being able to speedily diagnose a case of cholera will undoubtedly, in time, render a most valuable aid in checking its spread, and by taking the proper precautions after recognizing its presence, the danger of an epidemic will be greatly lessened. From a medical point of view, however, its utility at the present time is very slight, but it must be remembered that rational therapeutics for the majority of diseases, and especially for those of an infectious character, cannot be obtained until we have ascertained their precise causes. It is certainly to be hoped that the presence of the comma-bacillus may be of service in diagnosing Asiatic-cholera, and more especially so, in the early cases of its visitation. For the diagnosis, however, cultivation experiments are indispensable, and few have either the knowledge or the conveniences to enable them to carry this out. It is with a view of relieving the former of these wants that I have written this paper. No doubt, if Dr. Koch's theory is confirmed, some steps will be taken, in places threatened with an epidemic, to have means at hand for the satisfactory and rapid determination of the disease in suspicious cases. At present, if the discharges from suspicious cases were forwarded for examination to those who are interested in this work, much useful knowledge might be acquired, and an early intimation of its existence gained.

The method in itself is so easily understood that a physician possessing an ordinary knowledge of microscopical research would have little difficulty in cultivating, in the pure state, any bacillus with which he may be especially interested, and in a comparatively short time. The method is essentially the same as that employed in the cultivation of many of the different classes of bacilli known to us at the present time. Among these may be mentioned the typhus bacillus and tuberculosis bacillus, both of which are of recent discovery. A single week, perhaps, would be sufficient for developing and studying the peculiarities of any one species, but in order to appreciate minute differences, several species should be cultivated at the same time. In the course under Dr. Koch are cultivated, side by side, the Finkler-Pryor bacillus, the comma-bacillus, the typhus bacillus, besides several forms of micrococci, all to render stronger the contrast between them. The method of introducing, for example, the Finkler-Pryor bacillus and the comma-bacillus into the same re-agent glass, is also resorted to, and with the result of always finding their modes of development perfectly distinct one from the other. Nor is the same nourishing medium employed in all cases. Gelatine, bouillon, agar-agar, blood-serum and potatoes are all used as nourishing substances, and the various methods of preparing them will be explained further on.

The one precaution to be observed in bacteria cultivation is to thoroughly sterilize all vessels and instruments used in the promotion of the culture. This is effected either by a dry heat of 160° Centigrade, or a vapor heat of 100° Centigrade. The former is on all accounts the more satisfactory, although somewhat destructive to the fine tempering of steel instruments. The substance known as "food-gelatine" is most commonly employed as a breeding medium by the students in Dr. Koch's laboratory and its mode of preparation is as follows: Take 250 grams of fresh beef as free from fat as possible, and, after cutting it up into fine particles, add 500 grams of distilled water. Allow this to stand over night in an ice-chest or cellar and then strain it through a towel of ordinarily fine texture. The resulting mass will amount about to 400 *ccm.* Place the jar containing this substance in a metal vessel partly filled with water, and over a gas-jet allow it to reach the body-heat. Now add 40 grams of stick-gelatine, 4 grams of peptone and 1 gram of salt. It requires one-half hour for the gelatine to become thoroughly dissolved although this time may be somewhat lessened by occasionally stirring the mass with a sterilized glass-rod. The addition of a little carbonic acid will enable one to prove the reaction. For this purpose small pieces of red and blue litmus paper are used. Enough of the carbonic acid should be added to prevent the blue paper from changing color when a drop of the nourishing substance has been poured upon it. As a further test a single drop should cause the red paper to become blue in color. When this result is obtained, the whole mass is to be thoroughly cooked until it has the appearance of the white of an egg. In order to insure the utility of the entire mass, a little should now be strained into a sterilized re-agent glass and the reaction again be taken as above mentioned. If this proves satisfactory, the whole solution is to be strained through a double thickness of filter-paper arranged in the form of a funnel. Of course this process is an exceedingly slow one, and, if possible, it is best to have several funnels at work at the same time. The filtered substance is perfectly clear and transparent, and while still warm should be poured into re-agent glasses. These are prepared by first cleaning them and then closing their openings with wads of cotton. The process of sterilization is the same as that employed in other vessels, but the cotton-wadding, by turning slightly brown, enables one to tell very nicely when the glass is sterilized. It requires from forty to fifty re-agent glasses to hold the filtered mass, each one being filled to about one-third of its length. The idea of utilizing only a part of each tube will be better understood when the process of cultivation is well under way. After filling the requisite number of glasses and carefully replacing the cotton corks, they are to be placed together in a metal pot and boiled for the period of one hour. At the end of twenty-four, forty-eight and seventy-two hours respectively, they are to be again boiled for the period of three-quarters of an hour. We now have the medium in which all future cultivations can be carried on in the most satisfactory manner, and although certain characteristics may, perhaps, be better observed in some of the substances to be described further on, this food-gelatine is the one to which the greatest preference is given.

Another substance, which has considerable merit as a breeding medium for comma-bacilli, is agar-agar, or what is more commonly called "Ceylon moss." Its mode of preparation is similar to that of food-gelatine, except that one-half per cent. gelatine instead of ten per cent. is added. After being thoroughly cooked, it must be filtered through a double-walled hot-water funnel and then placed in the re-agent glasses. Agar-agar jelly is not liquefied by the colonies of comma-bacilli, and in this respect possesses a marked advantage over food-gelatine.

The cultivation of comma-bacilli in bouillon and on the cut surfaces of boiled potatoes will include a description of how these substances are prepared, and I will, therefore, proceed to explain the cultivation in food-gelatine. A small-sized platinum needle is the most convenient instrument with which to transfer materials of this kind, and after carefully sterilizing the point, remove from the contents of the intestine a single drop, so small as to be scarcely perceptible. Insert the needle into a tablespoonful of food-gelatine in liquid form, and shake it for a few seconds in order to thoroughly distribute the germs in the nourishing medium. From this tablespoonful take a platinum-pointful, and insert it into a second tablespoonful of gelatine, and in a similar manner one from the second into a third, always being careful to sterilize the needle before and after using it.

We now have three masses of gelatine, each inoculated with the cholera germ: the first directly from the excrement; the second indirectly from the excrement, having passed through one of the gelatinous masses; the third, apparently quite free from all germs, is still indirectly derived from the excrement, although having passed through two of the gelatinous masses. These are numbered, for convenience, 1, 2 and 3 respectively, and are to be poured upon three plates of ordinary window-glass for the purpose of cooling, and thus rendering them accessible for microscopical examination under a low power. A piece of glass eight inches long and six inches wide, well sterilized, will be found to serve the best purpose. Exposure to the cold causes the food-gelatine to become hard in a very short time, and the cholera bacilli, distributed through it, will begin to form colonies in the exact place where they are poured out. In order to prevent foreign matter from entering the gelatine before it has become hardened, the three plates are placed one upon another with an intervening bridge between them, and the whole covered with a bell-jar. Through this mode of development a perfectly safe diagnosis of the comma-bacillus may be made in from twenty-four to thirty-six hours. It moreover facilitates, in a marked degree, a further inoculation in firm, hardened gelatine, or in bouillon, and makes the preparation of colored microscopical specimens a comparatively easy task.

At the end of twenty-four hours the three plates should be examined under a magnifying power of 100 diameters. It has been my experience, during Dr. Koch's course, that in twenty-four hours' time only plate No. 1 gives any satisfactory indications of colony-formation, although I believe this depends somewhat upon the strength of the inoculation. In the early stages of its growth the colony resembles a small white spot upon the yellow background of food-gelatine; its form is nearly circular, with but very little symmetry on account of the rough and jagged appearance of its outline; the centre seems to be hollowed out, and here and there a small dark spot may be seen. A little later a very noticeable granulation of its contents takes place and certain changes in form and size easily distinguish it from colonies of other bacteria. With the gradual growth of the colony this granulation becomes more and more evident, and at last looks like a little mass of strongly refracting granules. During the more advanced stages, the gelatine in the immediate neighborhood of the colony undergoes liquefaction and causes the latter to sink much deeper into the gelatinous mass. A funnel-shaped cavity is thus formed, in which the colony is seen as a small whitish point. This appearance, according to Dr. Koch, is quite peculiar to the comma-bacillus. It is seen, at least, in very few other kinds of bacteria, but never shows itself in such a marked degree. The following cuts will serve to illustrate the various stages in the growth of the cholera colonies, as they appear upon the gelatine plate:

Youngest colonies.

More advanced.

After the gelatine has become liquid.

The sinking of the colonies can be better observed by carrying out an artificial cultivation. In order to do this, select a suitable colony, using a magnifying power of 100 diameters, and, with a fine platinum needle, well sterilized, remove from the colony a small drop and place it in a re-agent glass of food-gelatine. A cultivation of this kind then grows in the same manner as the colony on the gelatine plate. At the end of twenty-four hours a little funnel-shaped film marks the place of inoculation, with perhaps a slight extension of the film into the gelatinous mass. This increases more and more until finally the gelatine begins to liquefy around the point of inoculation. Then the little colony extends itself, and at the lower end of the film may be seen a deep spot which gives the appearance of an air-bubble hovering over the colony. Dr. Koch regards the air-bubble appearance as peculiar to the growth of the comma-bacillus, and as identical with the apparent cavity above the white spot on the gelatine plate. Any number of artificial cultivations can, of course, bé made from such a growth, but the same precautions must be observed in all cases in order to insure successful results.

The mode of cultivating the comma-bacillus in agar-agar jelly is the same as that employed in food-gelatine, and by following out the methods described for the latter a luxuriant growth can be obtained. The fact that the agar-agar is not liquefied by even the advanced growth of the colony renders this substance very valuable as a breeding medium. In potato-culture, however, an entirely different process is resorted to. The potatoes should be as fresh as possible, not mealy or in any way discolored, and with few eyes. Those having bruises or scratches that have penetrated the surface should not be used. After carefully washing them and cutting out the eyes, they are to be placed in a five per cent. solution of sublimate for half an hour. At the expiration of this time they are to be thoroughly cooked in a steam-pot. While they are cooling the preparator can spend the time profitably in sterilizing half a dozen knives with which to cut them open; he must also wash his hands, but more especially the thumb and first finger, in the sublimate solution. In cutting open the potatoes great care must be taken not to touch the cut surfaces with the fingers, nor should the same knife in any case be used twice. With cut surfaces up, the potatoes are placed in a bell-jar, lined with filter-paper, and saturated with sublimate solution. The inoculation should take place immediately after cutting the potatoes, and the method is the same as the one first described. The contents of the platinum point should be spread over the greater part of the cut surface, then inoculated from the first potato into a second and so on. During the growth of the comma-bacilli upon potatoes the appearance is the same as that presented by the bacilli of glanders. A thin, pulpy and somewhat brownish coating spreads over the entire surface; the brownish tint, however, is not so intense as in the bacilli of glanders. Comma-bacilli flourish best at a temperature between 30° and 40° Centigrade (86° to 104° Fahr.), although they can be cultivated in temperatures both higher and lower, but their growth is greatly retarded.

So far I have endeavored to explain the methods of cultivating the comma-bacilli so that they can be examined in colonies under a low magnifying power. No reference, however, has been made to the mode of preparing specimens for microscopical examination under a high power, and for studying the characteristic appearance of the organism itself. For these purposes a bouillon cultivation is the most satisfactory, although dry preparations can easily be made from the colonies as they appear on the gelatine-plate, or from the potato-culture just described. The bouillon should be fresh and free from all germs, and, before using, should be boiled. A peculiar kind of object-glass is employed for bouillon preparations; it is of the same size as the ordinary microscopic-slide, but the centre is hollowed out similar to the cavity of a table salt-holder, thus giving ample room for the growth of the colony. A little vaseline is spread around the edges of this cavity to enable the cover-glass to rest firmly over it. With a sterilized platinum needle place a drop of the bouillon in the middle of the cover-glass and inoculate it with a small drop taken from one of the colonies on the gelatine-plate. Then place the cover-glass over the cavity of the slide, taking care not to have it touch the sides. The vaseline keeps the air out and at the same time serves the purpose of Canada balsam or some other mounting medium. Several slides should be prepared in this manner and then placed in a cool room for twenty-four hours. They are now ready for examination with the Abbe artificial lighting apparatus and an oil-immersion objective. The appearance presented is that of a swarm of white particles in constant motion; the form is scarcely discernible; now and then, however, their length is seen to be greater than their breadth. An almost infinite number can be noticed, but their violent movements prevent the characteristic "comma" form from being detected. This is, to say the least, an unsatisfactory picture, but the only means of rendering it more real is to apply some artificial coloring substance such as fuchsin or methyl-aniline blue. From a single bouillon preparation some twelve or fifteen dry specimens can be made. This is effected by carefully removing the cover-glass and inserting a sterilized platinum point into the cultivation. The contents of the platinum point are spread upon a dry cover-glass and a drop of the staining fluid added. After washing off the superfluous coloring matter with distilled water, and mounting the preparation in Canada balsam, the best possible view of the comma-bacillus can be obtained. The following cuts are taken from preparations made during the ten days' course under Dr. Koch, and will serve to indicate the form and size of the bacilli as they appear under a magnifying power of 600 diameters :

Object-glass preparation, from bouillon cultivation. (*a*) Screw-shaped threads of bacilli, magnified 600 diameters, stained with fuchsin.

Object-glass preparation, cholera-dejecta several days old, showing "S" shaped bacilli, 600 diameters, stained with fuchsin.

To give the dimensions of comma-bacillus would, indeed, be useless, because only a very poor idea could be derived from the extremely small numbers which would be necessary to represent its length, breadth and thickness. To compare it, however, with some other well-known bacillus, such as the "tubercle," will enable the reader to form at least some notion of its size, and at the same time admit of a comparison as to form and general appearance. The comma-bacillus is about three-fifths as long as the "tubercle," but much thicker and more bulky. A very evident curve, similar to that of a "comma," is noticed midway between the two extremities, hence its name. Occasionally the curve is so marked that it resembles a semi-circle. Then, again, two bacilli may cling together, but in opposite directions, thus presenting the appearance of the letter "S." Often in artificial cultivations the comma-bacilli grow in wavy threads, as is seen in one of the above illustrations. The wavy appearance is peculiar to the comma-bacillus; straight threads, however, are frequently seen among other bacilli; for example, the "anthrax." Dr. Koch inclines somewhat to the theory recently brought forward, that the comma-bacillus is not a genuine bacillus, but only a transition form between bacilli and spirilla. By further investigation perhaps this question can be decided, but at present, it matters little to which class it belongs, so long as its death-causing property can be definitely established.

BERLIN, Jan. 6, 1885.

## Figures and Tables

**Figure f1:**
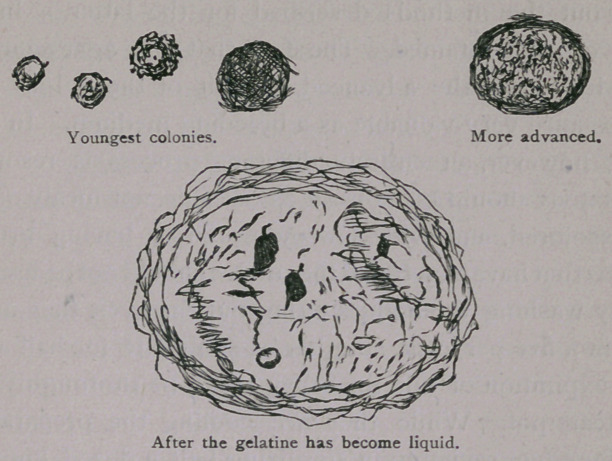


**Figure f2:**
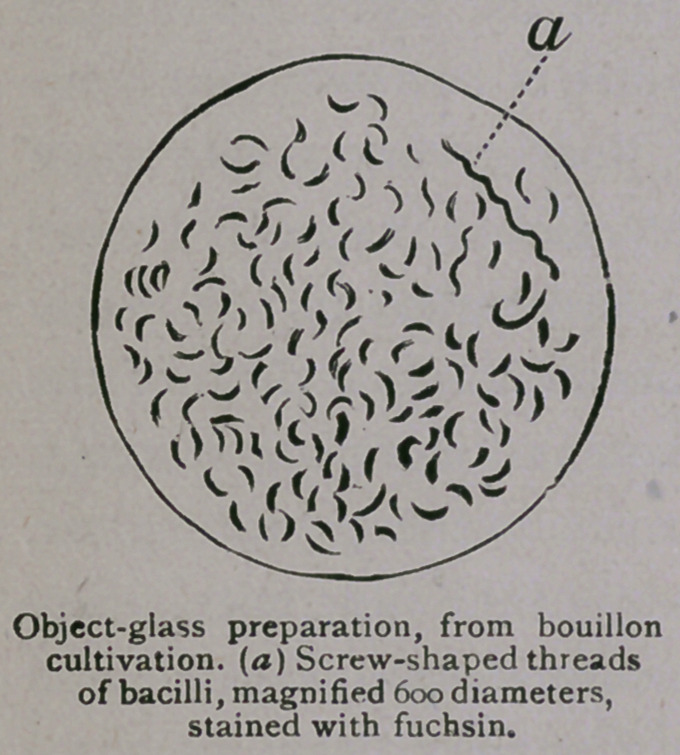


**Figure f3:**